# Femoral nerve block vs adductor canal block after anterior cruciate ligament reconstruction under general anesthesia: A prospective randomized trial protocol

**DOI:** 10.1097/MD.0000000000020776

**Published:** 2020-07-10

**Authors:** Qingpei Xue, Wei Jiang, Meng Wang, Jinkui Sui, Yiping Wang

**Affiliations:** a Department of Orthopedics. Qingdao Huangdao Central Hospital; b Department of Orthopedics. Laixi People's Hospital; c Department of Orthopedics. Qingdao Huangdao People's Hospital, Shandong, China.

**Keywords:** adductor canal block, anterior cruciate ligament reconstruction, femoral nerve block, pain control, randomized controlled trial, study protocol

## Abstract

**Background:**

Femoral nerve block (FNB) is considered the preferred analgesia after anterior cruciate ligament reconstruction (ACLR), but leads to weakness in the quadriceps muscles. Adductor canal block (ACB) is a new sensory block technique that effectively relieves postoperative pain while preserving quadriceps strength. The purpose of our study was to compare the efficacy of FNB vs ACB for pain control after ACLR.

**Methods:**

This prospective, randomized, double-blind, controlled, superiority clinical trial was approved by the institutional review board in our university hospital. We enrolled 120 patients set to undergo ACLR in this randomized therapeutic trial. Sixty patients received FNB and the other 60 received ACB for postoperative pain control. All ACB and FNB were performed using ultrasound-guided single-shot procedures. The primary outcomes included maximum voluntary isovolumetric contraction and postoperative pain score. Secondary outcomes included total opioid consumption, length of hospital stay, complication, and satisfaction score.

**Results:**

This clinical trial might provide some insights to estimate and compare the safety and efficacy of ACB vs FNB following ACLR.

**Trial registration:**

This study protocol was registered in Research Registry (researchregistry5569).

## 1 Introduction

Anterior cruciate ligament reconstruction (ACLR) has been increasingly employed in recent times to restore knee stability and optimize function and has been demonstrated as a safe, effective, and cost-saving procedure. However, patients often report moderate to severe postoperative pain requiring narcotic analgesia for pain control, especially within 24 to 48 hours after surgery.^[1,2]^ Great advances in pain management are well documented to be a major factor in the improvement of postoperative recovery after ACLR and the preemptive use of multimodal modalities is currently accepted as a principle of pain management after ACLR. As peripheral nerve blocks provide effective analgesia, they are considered an essential part of the current multimodal pain management protocol following ACLR.^[3,4]^

Given the excellent pain relief and synergistic analgesic effect, femoral nerve block (FNB) is commonly used as an analgesic modality and is considered the standard peripheral nerve block in patients undergoing ACLR. However, FNB is followed by a significant decrease in quadriceps muscle strength, resulting in delayed mobilization, which is associated with the potential risk of falling.^[5,6]^ Within this context, a growing body of evidence supports the use of an adductor canal block (ACB), which offers pure sensory block with minimal motor involvement in patients undergoing ACLR.^[7]^ An ACB can be expected to include the saphenous nerve, vastus medialis, medial femoral cutaneous, articular branches from the obturator, and the medial retinacular nerves. This distribution provides the innervation for the medial, anterior, and lateral portions of the knee.^[8]^

Some of the high quality studies have confirmed that ACB and FNB, in ACLR, can achieve similar postoperative analgesic effect, but patients with ACB can obtain better early rehabilitation compared with those with FNB.^[1–4]^ However, these studies suffer from several important methodologic shortcomings. Therefore, the question of potential analgesic and motor-sparing benefits of ACB in the setting of ACL reconstruction remains unanswered. Thus, this prospective randomized study was conducted to determine whether patients perceive a difference in pain level, and to investigate how different patients experience functional recovery of the quadriceps muscle with ACB and FNB after undergoing ACLR. Additionally, it was hypothesized that patients receiving ACB would exhibit similar postoperative outcomes compared with patients receiving FNB.

## 2 Materials and methods

**Figure 1:**
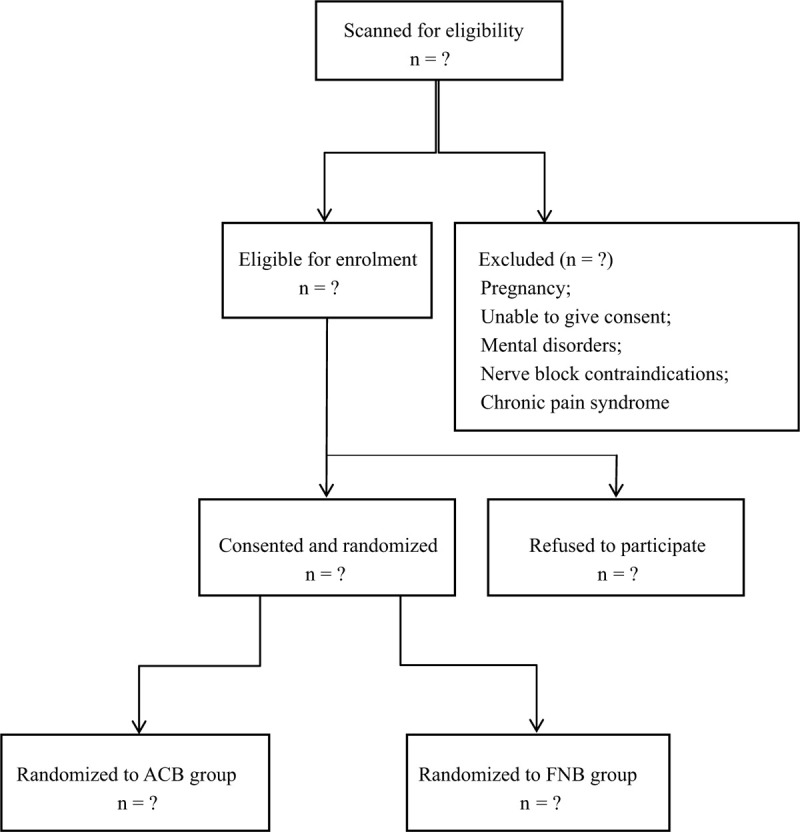
Flow diagram of the study. ACB = adductor canal block, FNB = femoral nerve block.

This blinded and randomized study was performed after approval of the institutional review board in our academic hospital (QHC2020071). It was carried out in accordance with the principles of the Helsinki Declaration. Data are presented according to the CONSORT statement. This trial was also registered in Research Registry (researchregistry5569). The flowchart of this trial is shown in Figure 1.

### 2.1 Study participants

Eligible patients included those aged <75 years, with an American Society of Anesthesiologists (ASA) score of 1 or 2, and who were scheduled for elective unilateral ACLR. Patients were excluded in case of pregnancy; significant psychiatric or mental disorders precluding assessment; baseline neuropathy or neurologic deficits involving the lower extremities; chronic pain or requirement of 30 mg or greater oxycodone (or equivalent) daily; nerve block contraindications, including coagulopathy or bleeding diathesis, local skin infections, and allergy to local anesthetics; or any component of multimodal analgesia. Patients who declined to participate in this trial or who were unable to provide informed consent were also excluded.

### 2.2 Randomization and blinding

After written informed consent was taken, patients were randomly allocated to one of the 2 groups in a 1:1 ratio using a computer-generated list of random numbers with a block randomization technique (www.randomization.com) by a research assistant. A unique randomization code was used with no restrictions to either of the 2 study groups: single dose or multiple doses dexamethasone. The results of the randomization were maintained in opaque envelopes and stored with the research coordinator. The patient, anesthesiologist, surgeon, physiotherapists, acute pain nurses, and outcome assessors were unaware of study group allocation.

### 2.3 Intervention

The ACB was performed at midthigh level during real-time ultrasonography using a linear high-frequency transducer placed intransverse orientation to visualize the femoral artery in short axis deep to the sartorius muscle. After the sterile field was achieved, the 21-gauge, 100-mm, short-bevel needle was inserted under ultrasound guidance in in-plane technique to position the needle tip anterolateral to the artery and just deep to the posterior fascia of the sartorius muscle. Once in position, 30 mL of investigative material was deposited adjacent to the femoral artery and deep to the sartorius muscle, using intermittent aspiration as described previously. After completion of the procedure, a sterile dressing was applied over needle insertion site.

The FNB procedure was conducted with the patient in a supine position, with the United States transducer applied to the skin at the level of the inguinal crease. The femoral artery, iliac fascia, and femoral vein were visualized. After the sterile field is achieved, the needle that is connected to the twitch monitor is inserted under ultrasound guidance using an in-plane technique from lateral to medial until a quadriceps motor response was elicited at a current between 0.5 and 0.2 mA with a pulse width of 0.1 millisecond from a twitch monitor. After negative aspiration, 30 mL of investigative material was deposited adjacent to the femoral nerve and deep to the iliac fascia, with intermittent aspiration. After completion of the procedure, a sterile dressing was applied over the needle insertion site. The success rate of the block was assessed by testing for sensation of cold in saphenous area of the lower leg by a trained physical therapy team at each time interval.

### 2.4 Outcome measures

The primary outcomes included maximum voluntary isovolumetric contraction and postoperative pain score. The maximum voluntary isovolumetric contraction, which measures quadriceps strength and is normalized to the body mass index (*N* ∗ m/kg). This test correlates well with the functional outcome. We placed the standard handheld dynamometer perpendicular to the tibial crest 5 cm proximal to the medial malleolus to make the measurement as previously described by Maffiuletti. The patients were told to “reach maximum force and hold for 3 seconds.” Three measurements were done, and the average was taken. The pain score was measured using visual analog scale (VAS) score (in the scale of 0 to 10, where 0 = no pain and 10 = worst pain that can be tolerated). VAS scores were recorded by nursing staff, blinded to treatment group, every 6 hours throughout the hospital stay.

Secondary outcomes included total opioid consumption, length of hospital stay, complication, and satisfaction score. Total opioid consumption was calculated by converting opioids consumed to morphine equivalents. Length of hospital stay was calculated by measuring the time from the completion of surgery through discharge for each patient.

### 2.5 Statistical analysis

All the data were analyzed using SPSS v. 24 (IBM Corp, Armonk, NY). We used the Kolmogorov–Smirnov test to assess whether variable distributions violated the assumption of normality. Data are presented as mean and standard deviation or with medians and 25th to 75th percentiles as appropriate. The normal distributed numerical variable (VAS scores, quadriceps strength, Range of motion, total opioid consumption, and patient satisfaction scores) was analyzed by Student *t* test. If the numerical variable has a nonnormal distribution or unequal variance, the Wilcoxon and Mann–Whitney *U* test was used (ASA grade); Pearson Chi-squared test or Fisher exact test was used to analyze the qualitative variable (inpatient falls). The nature of the hypothesis testing was 2-tailed, and a *P*-value <.05 was considered statistically significant.

### 2.6 Sample size calculation

The sample size was determined for the primary endpoint. According to the results of our previous study, the postoperative VAS score for nausea was 2.16 in the control group. We anticipated a difference of 0.72 in the VAS score. With a power of 0.90 and significance level of 0.05, the required sample size was calculated as 50 in each arm. Considering possible exclusion, we decided to include 60 patients in each group.

## 3 Discussion

The ACLR is one of the most frequently performed orthopedic procedures. The ability to perform ACL reconstruction on outpatient basis is largely dependent on an effective analgesic regimen that minimizes the role of systemic analgesics yet provides adequate postoperative pain control and eliminates the need for overnight stay or readmission.^[9]^ FNB is an effective analgesic technique for ambulatory ACL reconstruction; however, it weakens the quadriceps muscle, an outcome that is preferably avoided. In addition, recent evidence of persistent strength deficits in patients receiving FNB further underscores the need for effective alternatives.^[7]^

The ACB, a regional analgesic technique, is successfully used for postoperative pain control after knee surgery. In a retrospective cohort study, Manickam et al have demonstrated effectiveness of the ACB plus LIA on early ambulation after TKA compared with LIA alone.^[10]^ Some of the high quality studies have confirmed that ACB and FNB, in total knee arthroplasty, can achieve similar postoperative analgesic effect, but patients with ACB can obtain better early rehabilitation compared with those with FNB.^[5–9]^ Similarly, in a prospective randomized, controlled trial, patients who underwent ACLR were randomized to receive either FNB or ACB, ACB exhibited early relative sparing of quadriceps strength at 6 to 8 hours postanesthesia compared with FNB.^[1,11–15]^

The present research seeks to compare the effects of ACB and FNB on the postoperative analgesic area, analgesic effect, and early rehabilitation in patients undergoing ACLR. The main limitation is that the clinical effects and complications of ACB were compared with that of FNB without the control group in the present study. According to the beneficence principle for patients, no control group with placebo was designed in the protocol of the present study. We do not think this limitation would affect the results tremendously.

## Author contributions

**Conceptualization:** Qingpei Xue, Wei Jiang.

**Data curation:** Qingpei Xue, Wei Jiang.

**Formal analysis:** Qingpei Xue, Wei Jiang.

**Funding acquisition:** Jinkui Sui.

**Investigation:** Qingpei Xue, Wei Jiang.

**Methodology:** Yiping Wang.

**Resources:** Jinkui Sui.

**Software:** Qingpei Xue, Wei Jiang.

**Supervision:** Jinkui Sui.

**Validation:** Qingpei Xue.

**Visualization:** Qingpei Xue, Wei Jiang.

**Writing – original draft:** Qingpei Xue, Wei Jiang

**Writing – review & editing:** Meng Wang.
